# Cytokines in the colon, central nervous system and serum of irritable bowel syndrome rats

**DOI:** 10.1186/s40001-021-00479-w

**Published:** 2021-01-13

**Authors:** Guanqun Chao, Zhaojun Wang, Xinli Chen, Shuo Zhang

**Affiliations:** 1 Department of Family Medicine, Sir Run Run Shaw Hospital, Zhejiang University, Hangzhou, China; 2 Department of Gastroenterology, The First Affiliated Hospital, Zhejiang Chinese Medical University, Hangzhou, China

**Keywords:** Irritable bowel syndrome, Colonic mucosa, IL-1β, TGF-β1, CRF

## Abstract

**Objective:**

The aim of this study was to detect the expression of interleukin (IL)-1β and transforming growth factor (TGF)-β1 in the colonic tissue and serum of irritable bowel syndrome (IBS) rats, as well as the distribution and expression of corticotropin-releasing factor (CRF) in the spinal cord and brain of the visceral hypersensitivity rats, thus to ascertain the mechanism of visceral hypersensitivity signal conduction pathway.

**Methods:**

The expression of IL-1β and TGF-β1 in the colonic tissue and serum of IBS rats was screened by the liquid chip technology and verified by RT-PCR technology. Then the quantitative analysis of CRF in the spinal cord and brain was achieved by the immunohistochemical method and computerized image system.

**Result:**

The rat model with visceral hypersensitivity was successfully established. Among the screened indicators of IL-1β and TGF-β1 in colon tissue and serum, only the expression of IL-1β in the model group was up-regulated (*P* < *0.05*). The immunohistochemical method showed that CRF was expressed in the spinal cord, hypothalamus, and the third ventricle. The positive index number of the model groups was higher than that of the control group (*P* < *0.01*).

**Conclusion:**

From the research, it can be inferred that IL-1β may participate in the pathogenesis mechanism of IBS via regulating the colon function. The increasing expression of CRF linked to stress in the spinal cord, hypothalamus and the third ventricle indicated that it might play an important role in the mechanisms of visceral hypersensitivity signal conduction pathway.

## Introduction

Irritable bowel syndrome (IBS) is defined as a functional gastrointestinal disease without organic abnormalities and is characterized by chronic abdominal pain or discomfort [1]. Its incidence is about 10–20% in European and American [2], at least 6.5–10.1% in Asian and it has been continuing to grow in recent years [3]. Although IBS is one of the most frequent diagnosis in gastrointestinal diseases, the underlying mechanism of its chronic symptoms remains unknown [4]. Some factors like infection, food, and psychiatric factors have been stated as the possible etiology connected with the development of IBS [5]. It is clear that some IBS patients show remarkable inflammation and/or immunological disturbance in the colon [6]. Further studies revealed that transforming growth factor (TGF)-β1 could suppress the intestinal inflammation by modulating the growth and differentiation of different immunocytes and non-immunocytes [7]. And interleukin- (IL-)1β was reported to have the effect of regulating the initial stages of inflammation [8]. The study has shown that TGF-β1 and IL-1β are connected with inflammation, so we suspect they might play a role in the inflammation progression of IBS. Corticotropin-releasing factor (CRF) is the key regulatory factor in stress reaction. Some experiments indicated that the colon would be stimulated when injecting CRF in the cerebral ventricle or peripheral vein of rats, presented as the accelerated transmission of the colon and hasty excrementitious venting [9], which might be connected with IBS. Therefore, this study was to detect the expression of IL-1β and TGF-β1 in the colonic tissue and serum of IBS rats by the liquid chip and RT-PCR technology to find the relationship between them.

## Materials and methods

### Subjects

20 adult female SD rats, about 200 g of every rat, were divided into two groups randomly, 10 for the control group and 10 for the model group. The feeding environment was provided by the experimental animal center of Zhejiang Chinese Medical University, where the temperature was 22–24 ℃, humidity less than 60 percent, noise less than 50 decibels.

This study was reviewed and approved by the Medical ethics committee of Zhejiang Chinese Medical University, including any relevant details. And the rats in our study were sedated before being killed; all experiments in this study were performed following relevant guidelines and regulations.

## Experimental procedure and method

### Sectionalization

#### Control group

The condition of rats was observed after 2 weeks’ normal eating and drinking. Then the visceral sensitivity of normal rats was evaluated by abdominal withdrawal reflex (AWR).

#### Model group

IBS model rats were established under the guidance of previous studies, including conditioned stimulus and unconditioned stimulus [10]. The conditioned stimulus refers to the special odor of camphor ball, while the unconditioned stimulus needs rectal distention pressure (> 60 mm Hg (1 mmHg = 0.133 kPa)) combining with extremities constraint with their extremities and trunk fixed, rats were put into the camphor ball cage for 45 min. Meanwhile, catheters were inserted into the rectum of rats and fixed at the root of their tails. The distal end of the catheter balloon was 1 cm away from the anal. The volume of the filled balloon was 1.6 ml (hydrostatic pressure in the balloon more than 60 mmHg) and it lasted for 60 s, then intermittently exhausted 3 min and filled gas 10 times once. This was a stressful process. There was one stress process on the first day, the same process was performed on the second day at the same time. The conditioned stimulus was done on the fourth day. The completed stress process was repeated once on the fifth day. The same conditioned stimulus as the fourth day was performed on the sixth and eighth day. The rats were fed normally on the third and seventh day. Then visceral sensitivity of the rats was evaluated by AWR.

### Model authentication

AWR was used to evaluate rats' visceral sensitivity according to our previous studies [10]. First, an 8F urethral catheter was lubricated by liquid paraffin and inserted per rectum and fixed at the root of the tail. The distal end of the catheter balloon was 1 cm away from the anal. Then, the sacculus was filled with water gradually after rats accommodated the environment, and the water injection volume was recorded when the rats raised their abdomen and made the back like a bow. Rectal distention lasted for 30 s every time and repeated 3 times to take a mean number.

### Experimental sample

After a laparotomy incision, a segment of 2-cm-length colon tissue was removed and placed in Tyrode's solution which consists of a mixture of 95% O2 and 5% CO2, with the temperature held at 37 °C. Then douched the aorta with 500 ml normal saline and cut the auricle of the right atrium until the liver completely turned white. Then we perfused 500 ml fresh fixation fluid (0.1 mol/IL phosphate buffer with 4% PFA) through the aorta till the extremities and spinal column turned hard (40 min). Then we took the brain and spinal column and put them into the formalin solution quickly.

### Experimental procedure

Firstly, the total RNA was prepared, and the primer test and sample assay were also completed. Then we performed reverse transcription. After creating and setting up a plate document, we prepared the PCR reaction plate and run it, finally, we analyzed the result. The main procedure was that [10]: high-throughput sequencing was conducted on independent samples. Total RNA was extracted from colon tissues with TRIzol (Invitrogen, Gaithersburg, MD, USA) using the one-step method. After purification, RNA concentration was analyzed using Nanodrop (Nanodrop Technologies, Wilmington, DE) and quality testing was conducted using BioAnalyzer (Agilent Technologies, Palo Alto, CA). Small RNA was purified from total RNA to enrich molecules in the range of 16–30 nt, and then 3′ and 5′ linker sequences were attached SuperScript II reverse transcriptase to synthesize cDNA. After that PCR amplification was conducted. Luminex 100TM was used to detect the expression of IL-1β and TGF-β1 in the colonic tissue and serum of rats.

### Immunohistochemical evaluation

Two footwork immunohistochemistry technologies were used [10]: cautiously add, dropwise, 3 percent hydrogen dioxide on the tissue (spinal cord, hypothalamus, and the third ventricle of the cerebrum), incubating in dark for 15 min. After flushed by distilled water, the chips were put into the PBS balanced solution soaking 3 times for 5 min each time. AG dark was repaired. Drop 50-100 ml CFR antibody fluid on the tissue and make them incubate for 30 min in the ambient temperature. Next, we washed the chips with PBS balanced solution and soaked the chips in it 3 times for 4 min each time. Then we dropped an appreciable proportion diluted biotin labeling antibody (1% BSA–PBS to dilute) and incubated it for 30 min in the ambient temperature. After that, we washed the chips with PBS and soaked them in the PBS balanced solution 3 times for 5 min each time. Then we dropped 50-100 ml developer DAB fluid and incubated the chips for 5–20 min till they completely colorated. And then the tissue was washed by distilled water and successively dehydrated by 85%, 90%, 95%, 100%, 100%, 100% alcohol. Finally, the chips were put into the xylene solution 3 times for 5 min each time.

### Negative control

Replaced CFR antibody with PBS, the consequence was negative.

### Analytical method

The computer image analysis software (the Carl Zeiss of the Imaging Systems of the Carl Zeiss company) was conducted to analyze images. We took 10 high power campus visuals and 40 times object glass (400×) in the typical places successively. Then we analyzed the masculine expression by quantitative analysis and calculated photodensity.

### Statistical analysis

All data were expressed as mean ± standard. Statistical analysis was conducted by SPSS package. Two sample’s mean numbers were compared by the T-test. The significance level was *P* < 0.05.

## Result

### The change of the model

After modeling, there was no significant difference between the behavior of the two groups. Rats of the model group overreacted when they were frightened or performed by intragastric administration.

### Model authentication

AWR was used to authenticate the models, thus to compare the amount of water required to cause the same stress response in two groups. The rectum affusion amount of the model rats ((0.84 ± 0.23) ml) were lower than the control group ((1.33 ± 0.43) ml), and the difference between the two groups was statistically significant (*P* < 0.01). The results showed that the visceral sensitivity of the model rats was higher, indicating the success of the model building.

### The screening of cytokine in colon and serum

The expression of IL-1β and TGF-β1 in the colonic tissue and serum of IBS rats was screened by the liquid chip technology and verified by RT-PCR technology. The result of IL-1β and TGF-β1 is listed in Table 1. We found that the expression of IL-1β in the colonic tissue and serum of the model group was up-regulated (P < 0.05). However, there was no significant difference in the expression of TGF-β1 between the two groups (*P* > 0.05).

**Table 1:** The expression of IL-1β and TGF-β1 in two groups of irritable bowel syndrome rats

Marker	Control group ${\overline{\text{x}}} \pm {\text{s}}$	Model group ${\overline{\text{x}}} \pm {\text{s}}$
IL-1β (serum)	21.18 ± 3.60	28.45 ± 6.04*
IL-1β (colon)	1371.60 ± 276.57	1701.58 ± 345.12*
TGF-β1 (serum)	11,094.62 ± 4405.44	10,785.76 ± 1787.84^#^
TGF-β1 (colon)	854.47 ± 336.83	970.04 ± 357.46^#^

The expression of IL-1β and TGF-β1 in rats with IBS was detected using RT-PCR technology for quantity analysis. Correlations among the two groups were analyzed using SPSS24.0 software

^*^ *P* < 0.05

^#^ *P *> 0.05, vs. control group

### Dyeing result

**Figure1:**
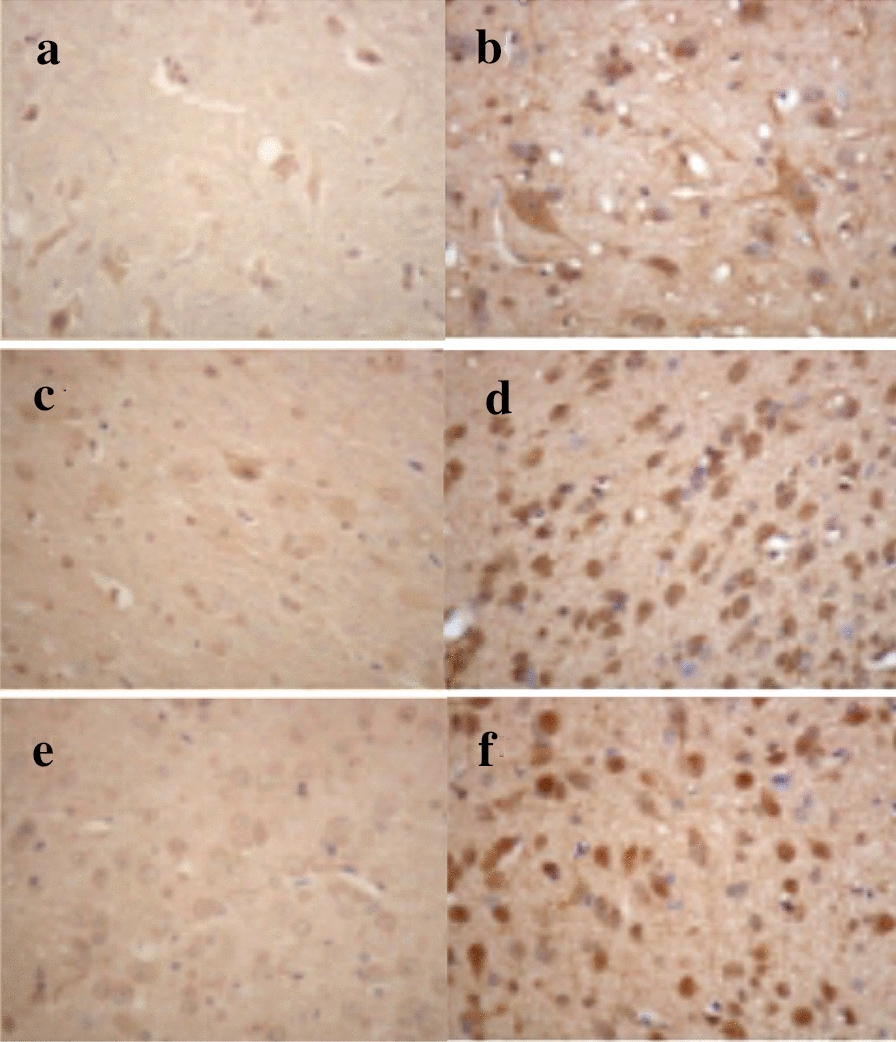
Immunohistochemical results of CRF in the central nervous system of the two groups. The brown particles refer to the expression of CRF. **a** The expression of CRF in the spinal cord in the control group. **b** The expression of CRF in the spinal cord in the model group. **c** The expression of CRF in the hypothalamus in the control group. **d** The expression of CRF in the hypothalamus in the model group. **e** The expression of CRF in the diaphragmatic surface of the third ventricle of the cerebrum in the control group. **f** The expression of CRF in the diaphragmatic surface of the third ventricle of the cerebrum in the model group

CRF in the spinal cord and brain was achieved by the immunohistochemical method and computerized image system. The positive areas of the chips were buffy. And they were more obvious in the hypothalamus, the third ventricle of the cerebrum, and spinal cord lumbar intumescentia, but weaker in another place. The typical positive neurons of CRF showed that the endochylema was buffy, and the nucleus could not be colored. We could find that positive neurons of CRF in different places have different shapes, such as fusiform shape, oval shape, polygon, and so on. The masculine neurons of CRF in the hypothalamus and the third ventricle of the cerebrum were close and uniform. Their endochylema was colored buffy and shaped like drops, while the nucleus could not be colored and some looked like vacuolus. The shapes were round, fusiform shape, oval shape, and so on. The masculine neurons of CRF in the spinal cord were uneven, and the endochylema was colored buffy and looked like drops, also the nucleus could not be colored and the number of fusiform shapes was more than others. Furthermore, the volume of the masculine neurons of CRF was bigger than that in the brain (Fig. 1).

### The expression of CRF

Hypothalamus: The positive area was showed, photodensity was high, and the positive exponent of the model group was higher than the control group (*P* < 0.01) (Table 2).

**Table 2:** Comparison of CRF masculine neuron of hypothalamus (expressed by ${\overline{\text{x}}} \pm {\text{s}}$)

Array	Positive area µm^2^	Photodensity	Positive exponent
A	2622.41 + 655.38	6472.61 + 1549.48	0.005 + 0.001
B	12,480.47 + 2290.70*	45,931.12 + 10,787.41*	0.037 + 0.009*

The expression of CRF masculine neuron of hypothalamus was detected using ratios of IOD/area, which was performed for immunohistochemical quantity analysis. Correlations among the two groups were analyzed using SPSS24.0 software

* *P* < 0.01, vs. control group

A: the control group; B: the model group

The diaphragmatic surface of the third ventricle of the cerebrum: The positive area was showed, photodensity was high, and the positive exponent of the model group was higher than the control group (*P* < 0.01) (Table 3).

**Table 3:** Comparison of CRF masculine neuron of the diaphragmatic surface of third ventricle of cerebrum (expressed by ${\overline{\text{x}}} \pm {\text{s}}$)

Array	Positive area µm^2^	Photodensity	Positive exponent
A	2764.40 + 435.14	6605.40 + 1155.27	0.005 + 0.001
B	9816.24 + 2393.63*	46,399.50 + 10,461.24*	0.038 + 0.009*

The expression of CRF masculine neuron of the diaphragmatic surface of third ventricle of cerebrum was detected using Ratios of IOD/area for quantity analysis. Correlations among the two groups were analyzed using SPSS24.0 software

^*^represents *P* < 0.01, vs. control group

A: the control group; B: the model group

The spinal cord: The positive area was showed, photodensity was high, and the positive exponent of the model group was higher than the control group (*P* < 0.01) (Table 4).

**Table 4:** comparison of CRF masculine neuron of spinal cord (expressed by ${\overline{\text{x}}} \pm {\text{s}}$)

Array	Positive area µm^2^	Photodensity	Positive exponent
A	1154.93 + 409.45	2671.58 + 1222.93	0.002 + 0.001
B	7824.93 + 1169.19*	33,814.07 + 9645.65*	0.028 + 0.008*

The expression of CRF masculine neuron of spinal cord was detected using Ratios of IOD/area for quantity analysis. Correlations among the two groups were analyzed using SPSS24.0 software

* P < 0.01, vs. control group

A: the control group; B: the model group.

## Discussion

IBS is a chronic, intermittent disease characterized by abdominal pain or discomfort without specific pathophysiologic factors [11]. According to the Rome III criteria, the global prevalence of IBS ranges from 1.1 to 29.2 percent and shows a female predominance [12]. More than one mechanism contributes to the development of IBS [13]. It’s known clearly that dysregulation of the autonomic nervous system, maladjustment of the hypothalamic–pituitary–adrenal axis (HPA), increased pain sensitivity, imbalanced gut microbial population, and altered enteric physiology participate in the pathophysiology of IBS [14]. In recent years, extensive attention has also been paid to the roles of inflammation and immunity in IBS. The intestinal inflammatory response to infection has been stated as a kind of remarkable defense response [15]. A large number of T cells in different lymphoid compartments were reported in IBS patients intestine [16]. And the activated T lymphocytes can produce many cytokines such as IL-1β, IL-10, and IL-17, which were closely related to the inflammatory process [15]. Moreover, some studies revealed that the level of IL-1β was much higher in IBS-D patients [17, 18]. This may have something to do with the inflammatory response caused by them [19]. And it is consistent with our results, in our present research, the IL-1β level in the colonic mucous membrane and blood of the IBS group was significantly increased, compared to those of the control group. As we have known, IL-1β is an important pro-inflammatory factor which can cause an inflammatory reaction and destruction on the mucosal barrier, or result in constriction and inflammatory pain for its direct action on mucosa and colonic smooth muscle [17]. However, some other researches reported that the expression of IL-1β showed no difference between the IBS group and the control group. We speculate that it may be related to the regulation of different T cells and the interaction between various cytokines. This network structure is worthy of further study to verify. At present, many studies confirmed that peripheral and local cytokines may affect GI function. Actually, not only cytokines and inflammatory reactions, but also psychological factors play pivotal roles in the development of IBS [20]. Our findings have also suggested that psychological status effects on the levels of cytokines, inducing or aggravating the digestive symptoms of IBS rats. Therefore, our further research is supposed to explore whether there is a cross-factor path to regulate cytokines such as IL-1β in IBS patients.

TGF-β1 is a member of the growth factor supper family. Stadnicki et al [21] reported that the high level of TGF-β1 was detected in the muscle of pro-inflammation type IBS, which could last for at least one month and effectively improve the excitability of smooth muscle. It was thought to contribute to the inflammation in IBS [22]. On the other hand, Gonsalkorale’s research [23] showed no significant difference in the TGF-β1 level between IBS rats and the normals, which was the same as our results. This may have to do with the fact that the production of cytokines is under genetic control. The genetic susceptibility of specific cytokines with gene polymorphism may change the sensitivity to certain diseases or affect their clinical expression [23]. What’s more, it should be noticed that many points need to be considered in the explanation of research findings. TGF-β1 is just one of the cytokines relevant to the regulation of immune and inflammatory, and the possible involvement of other cytokines in the process cannot be ruled out. Accordingly, the specific role of TGF-β1 in IBS still needs further study.

CRF is a kind of neuroendocrine peptide, which distributes in the stress-related important area of the central nervous system [24]. As a key regulatory factor in the stress reaction, CRF can produce a series of biological effects, such as removing the system to consent stress excitation, accommodating endocrine, autonomic nerve, immunifaction, and regulating behavioral response [25]. Combining with its receptor, CRF could keep gastrointestinal motility in a stringent state, which accelerates colonic transmission and causes watery diarrhea [26, 27]. During colonic dynamo-actuation, the nucleus of the hypothalamus and locus caeruleus were major locus to stimulate colonic movement. When microinjection of CRF was done to the nucleus of the hypothalamus and locus caeruleus of rats, colonic movement time decurtated, and the fecal output effectiveness could increase 20 times [28]. Correspondingly, when the injection of CRF was done to the centrum of the rats, visceral pain could aggravate; whereas, peripheral injection of CRF to mankind could degrade the pain threshold of the colon and increase bowel movement. Besides, some animal experiments found that injection of CRF to the center could cause turbulence of animals, such as gripping, scratching, and so on. As the dose increases, it could lead to spastic reactions, such as the increase of defecation and water sample stool [29]. This suggested that connections existed between psycho-behavioral changes caused by CRF with bowel movement. The latest clinical researches also pointed out that 57% of IBS patients had manifested visceral hypersensitivity. Based on the above discussion, this study aimed to investigate the distribution and expression of CRF in the spinal cord and brain of the visceral hypersensitivity rats and to reveal the key factor in the process of visceral hypersensitivity conduction.

The results showed that the level of CRF was quite high in the spinal cord, hypothalamus, and the third ventricle of the cerebrum. While low and sporadic CRF positive reactant distributed in neuronic endochylema. This stated that these three places all had neuroendocrine functions for physiological functions. CRF positive reactants in the model group were close, yellow, and deeper, with a positive index number increased obviously. It was clear that the expression of CRF increased in the models, and the visceral hypersensitivity was directly proportional to it. According to previous studies, the release of CRF was connected with emotional depression, and the special smell could induce depression. Importantly, CRF was expressed obviously in the hypothalamus, which suggested that central nervous system changes also occurred in visceral hypersensitivity. CRF and locus caeruleus–arterenol system might participate in the formation of visceral hypersensitivity besides the HPA axis. So we believe that CRF released in the hypothalamus could affect intestinal movement. The intestinal sensory receptor in visceral hypersensitivity could induce nervous excitation of CRF in the hypothalamus and other correlated areas, resulting in intestinal movement as well as externalization of corporal, which could explain the relationship between IBS and CRF. The mechanism might be that the release of CRF increased under a stimulative state, in turn, the released CRF could increase the releasing frequency of locus caeruleus, then induce neurofibrae projections to descend to myelonic sympathetic neuron and cause overexpression of CRF in the spinal cord. Therefore, locus caeruleus was the transition of power of bowel movement to cause visceral hypersensitivity. Through this experiment, we thought that CRF could be considered as an important factor to study the visceral hypersensitivity conduction system. Combining with many studies performed on the mechanism of visceral hypersensitivity, we confirmed that CRF could be an original target to treat IBS. At the same time, it would play an important role in the evaluation of the curative effect as well.

## Conclusion

This study suggested that increased CRF and IL-1β are involved in the mechanism of IBS. And CRF may be an original target in the pathogenesis of IBS. But further research need to be conducted to detect the interacting and interadjusting mechanism of IL-1β, TGF-β1, and other cytokines in IBS.

## Data Availability

The datasets used and/or analyzed during the current study are available from the corresponding author on reasonable request.
